# ELECTRONIC PERSONAL HEALTH RECORD USE AMONG ELDERLY CANCER SURVIVORS AND NON-CANCER SURVIVORS

**DOI:** 10.1093/geroni/igz038.1185

**Published:** 2019-11-08

**Authors:** Yan Luo

**Affiliations:** University of Alabama, Tuscaloosa, Alabama, United States

## Abstract

Background Electronic personal health records (ePHRs) are potential tools to improve clinical outcomes through increasing patients’ self-management. Although elderly people, especially elderly cancer survivors, is a growing population who can benefit from ePHRs, little is known about its utilization among the elderly, particularly among those diagnosed with cancer. Objective By applying Anderson’s Behavioral Model of Health Services Use, this study aims to examine and compare the associated factors with ePHRs use among elderly cancer survivors and non-cancer survivors. Methods The data collected from the 2018 Health Information National Trends Survey (HINTS) was analyzed. The level of access to ePHRs among the elderly were assessed. Predictors of ePHRs use among elderly cancer survivors and non-cancer survivors were compared by conducting multiple linear regression. According to Anderson’s Model, predisposing factors, enabling factors, and need factors were included in the statistical model. Results The overall use of ePHRs remained low among 577 participants (mean = .87, SD = 1.72, range from 0 to 4). Non-Cancer survivors reported lower ePHRs use (mean = .83, SD = 1.77, range from 0 to 4). Race/ethnicity, education, regular health care providers, health insurance, social support, and medical conditions were associated with ePHRs use among non-cancer survivors, while age, gender, social support, and self-reported health status were related to ePHRs use among cancer survivors. Conclusion This study suggests additional efforts to increase ePHRs utilization among the elderly, especially the elderly cancer survivors. The predictive findings reported in this study will contribute valuable implications to enhance the ePHRs use.

